# Evidence of Effectiveness of Lingual Orthodontics as an Alternative to Conventional Labial Orthodontics. A Systematic Review

**DOI:** 10.7759/cureus.51643

**Published:** 2024-01-04

**Authors:** Suhael Ahmed, Rawda Alghabban, Abdulaziz Alqahtani, Khalid Alrehaili, Abdullah Aljarullah, Abdulaziz S Alghannam, Abdullah M AlHathlol

**Affiliations:** 1 Maxillofacial Surgery, Riyadh Elm University, Riyadh, SAU; 2 Orthodontics and Dentofacial Orthopaedics, Prince Sattam Bin Abdulaziz University, Al-Kharj, SAU; 3 Dentistry, Security Forces Hospital, Riyadh, SAU; 4 Dentistry, King Saud bin Abdulaziz University for Health Sciences, Riyadh, SAU; 5 Dentistry, Batterjee Medical College, Jeddah, SAU; 6 General Dentistry, King Saud bin Abdulaziz University for Health Sciences, Riyadh, SAU

**Keywords:** anterior aesthetics, braces, alternative orthodontics, incognito, lingual orthodontics

## Abstract

In orthodontics, both the treatment goals and the impact of orthodontic equipment on patients' aesthetic appearance have contributed to a rise in patients' aesthetic demands over the years. Patients considering orthodontic treatment are significantly concerned about the potential compromise in facial appearance that conventional orthodontic therapy might cause. Clinical practice has integrated aesthetic materials and procedures to address these restrictions. This review will examine the present data and outcomes linked to lingual orthodontics. PubMed, Scopus, Web of Science, Cochrane Library, and Embase were the electronic databases searched. Research interests mainly included biomechanics, appliance design, bonding, laboratory settings, case reports, survey research, and treatment outcomes. The goal was to locate the most recent data regarding lingual orthodontics. A consistent and predictable pattern emerges from the available evidence on lingual orthodontics. Several areas have received a lot of attention over the past decade, including the ability to forecast outcomes and patients' preparedness to embrace these changes. The current state of knowledge on the biomechanical principles of lingual orthodontics is solid, as this review shows. Lingual orthodontic appliances can efficiently handle any orthodontic scenario that a labial appliance can handle. The reason is that the completely customized lingual appliance might bring about the desired result in terms of treatment.

## Introduction and background

Over the years, patients have become more demanding regarding the aesthetic aspects of orthodontic treatment. This includes the treatment goals and the impact that orthodontic equipment has on the patient's appearance. Research has demonstrated that traditional orthodontic treatment can, at times, negatively impact the psychology and confidence of the patient, which is a significant worry for those seeking orthodontic care [1]. To address these constraints, clinicians have implemented aesthetic materials and procedures in clinical practice [2]. Lingual orthodontics represents a primary manifestation of this requirement. Since its inception in the 1970s, patients and physicians have increasingly shown interest in lingual orthodontics, leading to the development of numerous methods and approaches. An attractive feature of lingual orthodontic treatment is that the brackets are undetectable. However, the positioning of the appliances is what primarily distinguishes lingual orthodontics from established labial orthodontic therapy [3,4]. Lingual orthodontics can't be used all the time in regular orthodontic practice because of differences in treatment characteristics caused by the practitioner, the patient, or the appliance [5]. A multitude of case reports and clinical studies have investigated various facets of lingual orthodontic treatment throughout the years [6-8]. This study will assess existing information on the efficacy of lingual orthodontic treatment and its associated clinical factors.

## Review

Methodology

This review followed PRISMA principles for systematic reviews. After searching PubMed, Scopus, Cochrane Library, Embase, and Web of Science for "lingual and labial orthodontics," "lingual patients," and "lingual brackets," we evaluated twelve studies that met the inclusion criteria. We searched for post-January 2000 studies. Data extraction forms, eligibility evaluation, and search and analysis procedures were predetermined based on pilot project findings.

Eligibility Criteria

Inclusion criteria: This study included case reports, prospective controlled trials, and retrospective studies with the control group on the effects of fixed lingual bracket orthodontic therapy on bonded teeth between the first molars in one or both arches. Only English-language articles were considered.

Exclusion criteria: We excluded controlled laboratory and animal research, case reports, case series, editorials, comments, evaluations, and procedures without sample data. Also excluded was research on the lingual treatment of particular teeth in an arch, alone or with labial braces.

Control

Conventional treatment options include using labial fixed appliances or no treatment at all. The research also examined the expected treatment outcomes and compared patients who received fixed appliance therapy using different lingual bracket systems or application procedures, such as various bonding methods.

Types of Outcome Measures

We assessed the intervention's efficacy using precision and other treatment quality indicators, including periodontal and oral health. Treatment length, end-outcome stability, bond failures, and other clinical factors are considered.

Quality Assessment of the Included Studies

Two authors conducted the quality evaluation of the eligible studies independently. All writers reached a consensus through discussions to resolve areas of disagreement. The Cochrane risk-bias tool was utilized to determine the quality of randomized studies. Furthermore, the same instrument was used for non-randomized prospective investigations in relevant fields.

Results

**Figure 1:**
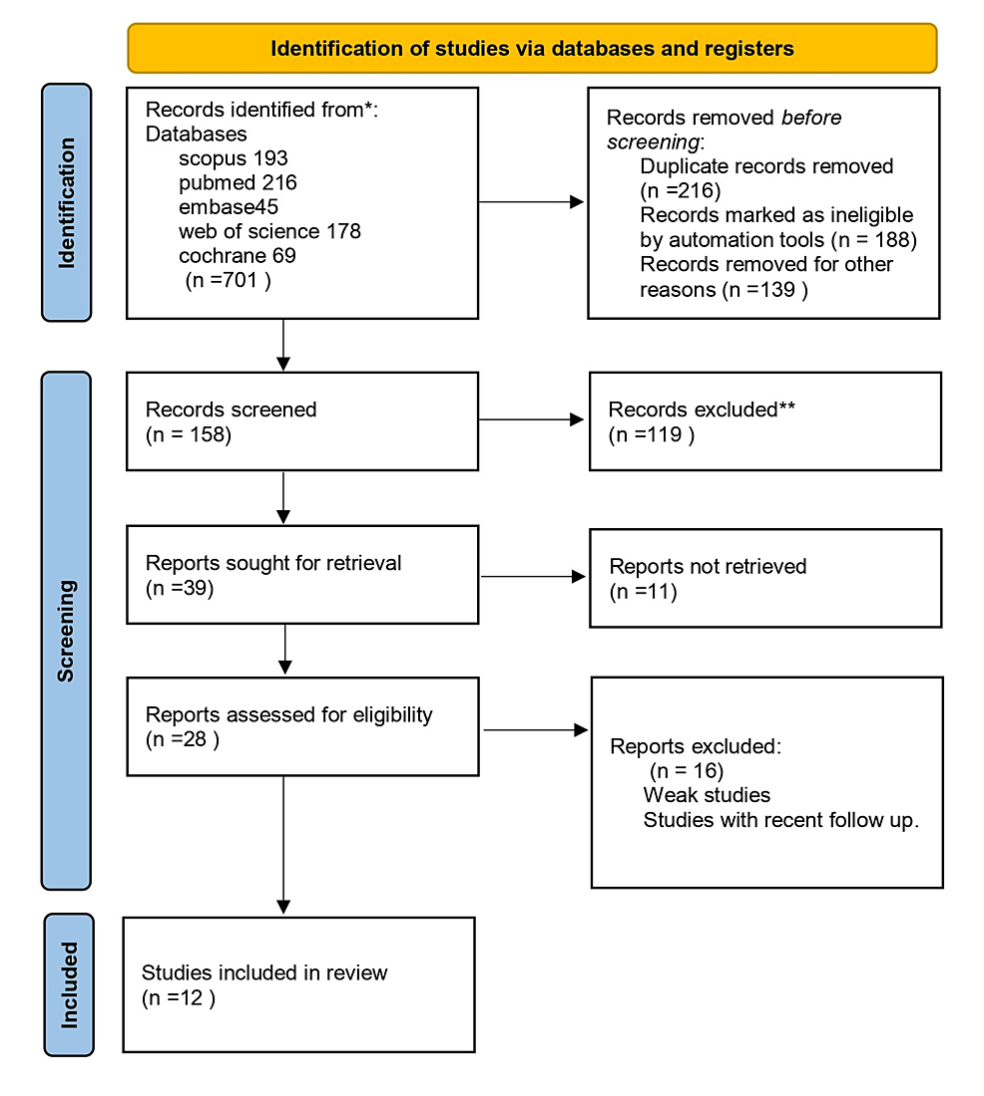
PRISMA 2020 Flow Diagram

A total of 701 studies were acquired during the initial search. After carefully examining the titles, inclusion and exclusion criteria, and abstracts, we comprehensively analyzed 158 studies. A total of 12 studies met the inclusion criteria for the review. Figure 1 presents a flow chart that visually represents the research selection process, including the explicit rationales for eliminating particular studies.

The Assessment of the Risk of Bias in the Included Studies

**Figure 2:**
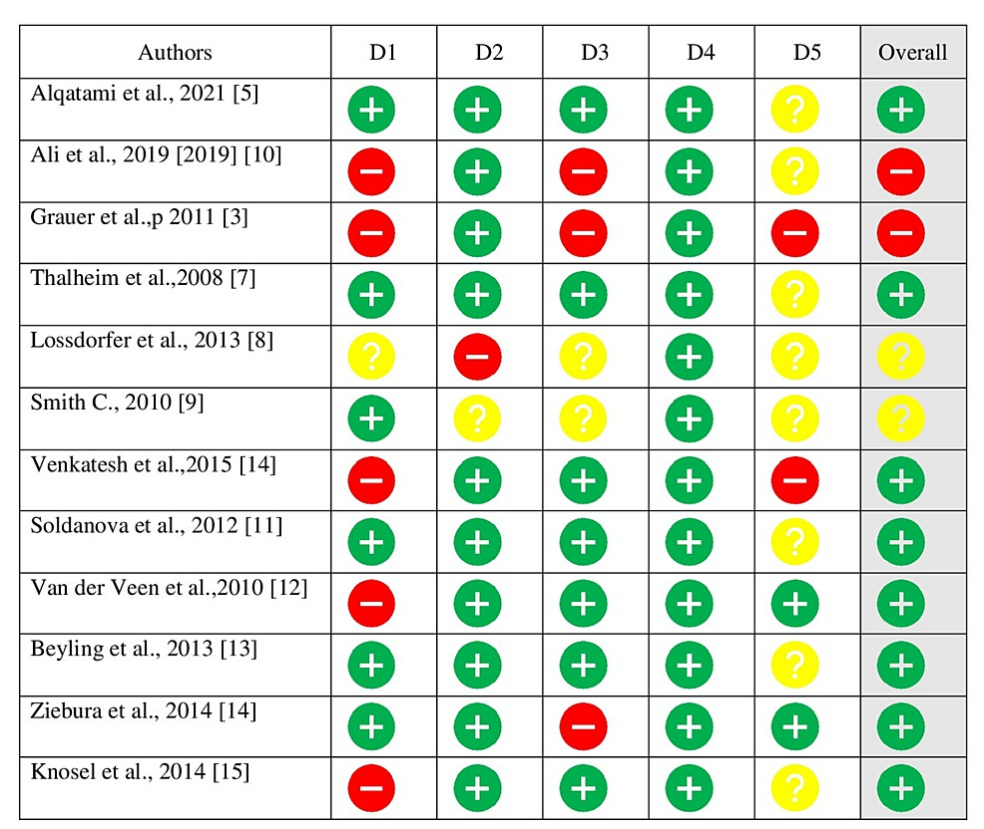
Risk of bias assessment with the recommended approach of Cochrane ROB 2. D1- Bias resulting from the randomization process, D2- Bias resulting from a departure from the intended interventions, D3- Bias resulting from missing data outcomes, D4- Bias resulting from faulty measurement of the outcome, and D5- Bias resulting from the selective reporting of results. Risk of bias is indicated by the colors red (high), yellow (some), and green (low).

RCTs: We incorporated only one randomized controlled trial [RCT] and evaluated its overall risk of bias as indeterminate. Figure 2 presents a concise overview of the risk of bias evaluation as per the Cochrane Risk of Bias methodology. One controlled clinical trial had an ambiguous assessment of bias, while the other had a high overall risk of bias. Figure 2 presents a concise overview of the risk of bias evaluation for non-randomized prospective controlled clinical trials [CCTs]. Two of the nine analyzed retrospective studies were determined to have a high overall risk of bias, whereas two studies had an unclear overall risk of bias. Figure 2 displays the evaluation of potential bias in retrospective research.

Characteristics of the Included Studies

Nine retrospective, two prospective controlled clinical trials (CCTs), and one randomized controlled trial (RCT) were the characteristics of the selected studies. Eight retrospective studies compared actual and planned lingual appliance treatment results to determine precision. Additionally, occlusal results between lingual therapy and labial therapy were compared. Seven studies assessed treatment efficacy, anchoring loss, binding failures, and duration. Two potential controlled clinical trials [CCTs] examined how treatment affected the gingival index and plaque index. Table 3 summarizes the studies' main features.

**Table 1:** Summary of key features of the included studies CCLA: complete customized lingual appliance; CCT: controlled clinical trials; RCT: randomized controlled trial

Study	Primary objective	Location of sample collection	Study Design	Sample size	Sample recruitment method
Alqatami et al., 2021 [9]	To evaluate the efficacy of subjects who underwent treatment using a complete customized lingual appliance (CCLA)	Germany	Retrospective single-arm study	10	Specific criteria-based selection
Ali et al., 2019 [10]	To compare the efficacy of orthodontic treatment among patients who received lingual appliance treatment and those who received labial appliance treatment.	Spain	Retrospective single-arm study	72	Double blinding
Grauer et al., 2011 [3]	The accuracy of the lingual orthodontic technique was determined	Germany	Case series/Retrospective study	Lingual treatment (Incognito) (n = 94)	Patients debonded between March 2009 and May 2010
Thalheim et al.,2008 [11]	Lingual orthodontic technique efficiency was determined	Germany	Case series/Retrospective study	Incognito lingual treatment (n = 20)	Specific criteria-based selection
Lossdorfer et al., 2013 [12]	The precision of the incognito lingual orthodontic technique was assessed.	Germany	Case- series/Retrospective study	lingual treatment (Incognito) (n = 34)	Specific criteria-based selection
Smith C., 2010 [13]	The accuracy of the lingual orthodontic technique (incognito) was assessed, along with the occlusal outcome and duration of treatment utilizing labial and lingual appliances.	USA	Case series/Retrospective study	lingual (Incognito) (n = 21) vs various labial (n = 22)	
Venkatesh et al.,2015 [14]	Anchorage loss during space closure in premolar region with labial and lingual appliances	India	CCT/ Prospective study	STB lingual (n = 10) vs labial (3M Unitek n = 10)	Double blinding
Soldanova et al., 2012 [15]	Mandibular dental arch modifications utilize the labial or lingual two-dimensional technique.	Czech republic	CCT/ Prospective study	lingual 2 dimensional ( n = 25) versus labial (Minitrim n = 25)	Specific criteria-based selection
Van der Veen et al.,2010 [16]	The prevalence of dental caries subsequent to lingual or labial appliance treatment	Germany	RCT [split mouth]	lingual (Incognito) (n = 14) vs labial (Ormco; n = 14)	Specific criteria-based selection
Beyling et al., 2013 [17]	To evaluate the impact of incorporating an additional hydrophilic resin layer during the bonding process, specifically in demineralization below the lingual bracket base.	Germany	Case series/Retrospective study	lingual bonded conventionally (Incognito) (n = 20) versus bonded with extra resin layer (n = 20)	Double blinding
Ziebura et al., 2014 [18]	To analyze and contrast the occurrence and location of bond failures in patients undergoing orthodontic treatment with buccal or lingual appliances.	Germany	Case series/Retrospective study	lingual (Incognito) (n = 59) versus Mini Diamond Brackets/ labial (n = 44)	Double blinding
Knosel et al., 2014 [19]	To assess the difference in treatment time between two distinct variations of personalized lingual equipment	Germany	Case series/Retrospective study	lingual (Incognito) (3M Unitek) (n = 220) vs WIN lingual Systems (n = 156)	Specific criteria-based selection

Discussion

The increasing quantity of pertinent studies in the literature reflects the scientific community's growing interest in lingual orthodontics. The review lasted for 23 years (2000-2023), and within the past 5 years, 4 out of the 12 articles included in the analysis were published. This emphasizes the need for orthodontic treatment to accommodate the increased aesthetic demands desired by patients. However, contemporary orthodontic practice does not widely regard the lingual orthodontic treatment option as a traditional choice. Possible reasons for this may include the limited understanding of the technique's clinical effectiveness, the need for specialized knowledge to implement it, the lack of comprehensive instruction on lingual orthodontics in most advanced training programs compared to traditional buccal techniques, and the typically higher associated costs.

In order to align with current clinical conditions, we chose to incorporate recent studies exclusively by implementing a temporal constraint in our search approach. Alternatively, using previous research may yield outcomes that are not relevant to current clinical practice due to the progression of lingual appliances and procedures throughout the years.

We categorized the 12 comparative studies into three broad groups based on the issue of investigation. We evaluated these factors: accuracy and clinical parameters-periodontal parameter testing.

Four retrospective investigations within the initial group compared the intended treatment objective outlined in the setup with the actual results achieved after treatment to assess the precision of lingual orthodontic treatment. Researchers compared the intended treatment objective outlined in the setup with the actual results achieved after treatment. The findings of these investigations were promising, suggesting that contemporary lingual orthodontic systems can effectively accomplish the personalized treatment objectives stated by the setup. Unfortunately, due to this research's retrospective nature, it is impossible to determine the specific factors that affect the success of the desired treatment outcome for each patient. In order to do this, it is imperative to conduct meticulously planned and carefully executed clinical studies.

The second subject group comprised seven studies that examined different clinical features of lingual orthodontic treatment. The only available randomized controlled trial (RCT) showed that the number of cavities in the back of the mouth that got worse during fixed buccal appliances was about five times higher than the number of cavities in the front of the mouth that got worse during fixed lingual appliances. However, the split-mouth study design made blinding infeasible, thus leaving open the possibility of performance bias throughout the study period. In order to validate the good impact of lingual appliance treatment on oral health, it is imperative to conduct further randomized controlled trials [RCTs] with substantial sample sizes. A further study examined the occurrence of demineralization beneath the bracket base on the underside of the tongue. Including hydrophilic resin during the bonding process significantly decreased the incidence of demineralization in children and teenagers [17]. The remaining investigations in the second subject group examined a range of topics, such as efficacy, duration of therapy, loss of anchoring, and failures in bonding. Researchers conducted a small-scale retrospective study to assess how the lingual technique and Herbst affect the forward migration of mandibular incisors [20]. The study positioned the incisors by the intended movement [20]. Another study that looked back found that lingual [Incognito] and labial appliances had similar effects on how teeth fit together and the length of treatment [21]. Two studies looked at anchorage loss in premolar extraction cases and compared lingual [STb or bidimensional, Ormco] and labial appliances. The results revealed that labial treatment resulted in nearly twice the amount of anchorage loss compared to lingual treatment [10, 18]. Researchers looked into the future and found that Forestadent 2D therapy, which involves moving the lower dental arch's labial and lingual parts, mostly has the same effect on people with Class I crowding [22]. A study that looked back at the past found that using lingual [STb] and labial appliances leads to similar decreases in PAR, treatment times, and root resorption levels [19]. A separate investigation revealed that the incidence of misplaced brackets within the initial year of therapy was comparable across lingual and labial appliances, with a total of 32 cases. A recent retrospective study conducted a comparison between two different lingual systems, demonstrating that the treatment period was much shorter with the WIN system compared to the Incognito system [19]. However, while interpreting the results of this research, it is important to consider the potential influence of numerous biases on the study outcomes.

The third subject group comprised two prospective controlled clinical trials [CCTs] that investigated the effects of lingual orthodontic therapy on several periodontal indicators. The original study included a split-mouth strategy to investigate the immediate effects of Incognito lingual appliances on various clinical periodontal and microbiological indicators. The appliances attached to specific regions showed an aggravation of the plaque index and bleeding on probing. This finding was also seen in other studies [23-25]. However, no significant discrepancy was found in the probing depth measurement or the presence of periodontopathogenic bacteria [18]. The other study investigated the effects of Ormco's seventh-generation lingual treatment and Invisalign treatment on clinical periodontal indicators. The indicators, except for probing depth, showed considerable improvement in the Invisalign patients during the assessments. Several case reports of successful treatment using the Herbst appliance in combination with lingual orthodontics showed good periodontal health [26-28]. However, in the lingual treatment group, all indices were considerably worse at all periods [13]. Regarding analyzing the results from these two investigations, it is important to note that both are individual, non-randomized studies with an uncertain level of bias.

Limitations

Consequently, most of the studies included in the analysis had a retrospective design, which resulted in a significant risk of bias. An evaluation indicating a high risk of bias does not automatically imply that the study findings are biased. Instead, it suggests a greater likelihood of biased outcomes compared to a study categorized as having a low risk of bias.

Due to the limited availability of studies, synthesizing the findings was generally impossible since each subject had only one study conducted. Due to the clinical heterogeneity of the included research, for example, testing different bracket systems, it was impossible to aggregate or synthesize the data and conclusions of multiple studies on the subject.

## Conclusions

This comprehensive study demonstrated promising findings regarding the clinical outcome of lingual orthodontic treatment, particularly regarding the decrease in decalcifications on the bonded tooth surfaces and the successful achievement of personalized treatment objectives. However, more clinical trials with larger samples may be necessary to confirm those results. Study design constraints, sample variability, small sample numbers, and considerable bias in most of the included studies made evaluating various aspects of lingual orthodontic therapy difficult.
